# Supplementary material for: Spatial equity in the layout of urban public sports facilities in Hangzhou
Source: PLoS One. 2021 Sep 2;16(9):e0256174. doi: 10.1371/journal.pone.0256174 (PMC8412330; doi:10.1371/journal.pone.0256174)
Supplement: Attachment — Submitted filename: 20210616Response to Reviewers.docx [file pone.0256174.s001.docx]

Dear Editor:

We feel great thanks for your professional review work on our article. As you are concerned, there are several problems that need to be addressed. According to your nice suggestions, we have confirmed and modified the following problems.

1) Thank you for providing the following statement in your Response to Reviewers document:

"Issues 3: We note your current data availability statement: "All relevant data are within the manuscript and its Supporting Information files." We also note your statement in your response to reviewers document: The paper data has been modified to be shareable.

Before we proceed, we’ll require some additional clarification to ensure your submission adheres to the PLOS ONE Data Availability policy (https://journals.plos.org/plosone/s/data-availability).

1) If you have upload your data to a repository, please also provide the relevant URLs, DOIs, or accession numbers for other researchers to access your data directly. For a list of recommended public repositories, please see <https://journals.plos.org/plosone/s/recommended-repositories>.

Response：The data of this paper has been uploaded to the repository, and the URLs is https://figshare.com/s/c05872042227c1a5d9fc. If necessary, please click on the website to visit.

2) Please clarify whether you intend to make your data available publicly upon acceptance for publication. Once we receive this information, we will update your data availability statement on your behalf.

Response：Data for this paper will not be made available publicly upon acceptance for publication."

Please note that PLOS only permits data to be restricted from public access if there are ethical or legal restrictions on sharing the data. Before we proceed, we’ll require some additional information to ensure your submission adheres to the PLOS policy on acceptable data access restrictions: https://journals.plos.org/plosone/s/data-availability#loc-acceptable-data-access-restrictions.

1) Please confirm whether there are legal or ethical restrictions on sharing your data publicly.

2) If legal or ethical restrictions apply, please provide all necessary instructions and non-author contact information (preferably email) for a data access committee, ethics committee, or other institutional body other researchers would require to apply for data access. Note that it is not acceptable for an author to be the sole named individual responsible for ensuring data access.

3) If there are no legal or ethical restrictions on sharing your data publicly, please upload the minimal anonymized data set necessary to replicate your study findings as either Supporting Information files, or to a stable public repository. If you upload your data to a repository, please also provide the relevant URLs, DOIs, or accession numbers for other researchers to access your data directly. For a list of recommended public repositories, please see https://journals.plos.org/plosone/s/recommended-repositories.

4) We note that the DOI provided (10.6084/m9.figshare.14754552) does note direct to your data set(s). If there are no legal or ethical restrictions on sharing your data publicly, please ensure that any URLs, DOIs, or accession numbers you provide direct to your data.

Response：There are no legal or ethical restrictions on sharing our data publicly. The URLs is https://figshare.com/s/c05872042227c1a5d9fc. We have chicked that the URLs directs to the data.

Thank you again for your positive comments and valuable suggestions to improve the quality of our manuscript !
